# Scintigraphic Small Intestinal Transit Time and Defaecography in Patients with J-Pouch

**DOI:** 10.3390/diagnostics5040399

**Published:** 2015-10-10

**Authors:** Mie Dilling Kjaer, Jane Angel Simonsen, Svend Hvidsten, Jens Kjeldsen, Oke Gerke, Niels Qvist

**Affiliations:** 1 Department of Surgery, Odense University Hospital, 29 Sdr. Boulevard, 5000 Odense, Denmark; E-Mails: niels.qvist@rsyd.dk; 2 Department of Nuclear Medicine, Odense University Hospital, 29 Sdr. Boulevard, 5000 Odense, Denmark; E-Mails: jane.simonsen@rsyd.dk (J.A.S.); svend.hvidsten@rsyd.dk (S.H.); oke.gerke@rsyd.dk (O.G.); 3 Department of Medical Gastroenterology, Odense University Hospital, 29 Sdr. Boulevard, 5000 Odense, Denmark; E-Mail: jens.kjeldsen@rsyd.dk; 4 Centre of Health Economics Research, University of Southern Denmark, Campusvej 55, 5230 Odense, Denmark

**Keywords:** scintigraphic small intestinal transit time, scintigraphic defaecography, ileal pouch-anal anastomosis, IPAA, ulcerative colitis

## Abstract

Objective methods for examination of pouch function are warranted for a better understanding of the functional result and treatment of dysfunction. The objective of this study was to evaluate the results of scintigraphic intestinal transit time and defaecography compared to the results of pouch function, mucosal condition and a questionnaire on quality of life (QoL). This cross-sectional study included 21 patients. Scintigraphic transit time and defaecography was determined with the use of Tc-99m. Pouch function was assessed by number of bowel movements, pouch volume, and continence. Pouch mucosal condition was evaluated by endoscopy and histology. Median transit time was 189 min (105–365). Median ejection fraction at defaecography (EF) was 49% (3–77) and 62% (17–98) after first and second defecation. Median pouch volume was 223 mL (100–360). A median daily stool frequency of nine (4–25) was reported and three (14%) patients suffered from daytime incontinence. No patients had symptomatic or endoscopic pouchitis; however, the histology showed unspecific inflammation in 19 (90%) patients. There was no correlation between transit time, evacuation fraction (EF) and pouch function in univariate analysis. However, we found a high body mass index (BMI) and a low bowel movement frequency to be associated with a longer transit time by multivariate analysis. Scintigraphic determination of transit time and defaecography are feasible methods in patients with ileal pouch anal anastomosis, but the clinical relevance is yet doubtful.

## 1. Introduction

Proctocolectomy and subsequent reconstruction with an ileal pouch-anal anastomosis (IPAA) for ulcerative colitis aims to eliminate the disease, avoid a permanent stoma, and result in acceptable pouch function along with good quality of life (QoL). The definition of pouch function is very heterogeneous, and can include various aspects, such as stool frequency, urgency, incontinence, incomplete emptying, use of pads, and anti-diarrheal medication.

A recent systemic review found a mean (95% CI) stool frequency of 5.9 (5.0–6.9) per 24-hour within 5132 adult IPAA patients and severe daytime incontinence in 6.1% (2.9–12.3) among 3718 patients [1]. A new Swedish study concluded that more than 35% of patients had a stool frequency of six or more regardless of pouch design and type of anastomosis [2]. Stool frequency varies from day to day. Patients’ perception of problems with frequent bowel movements also varies; some may easily tolerate more than 10 stools per day.

Several self-reporting scoring systems exist to evaluate the pouch function [3,4,5], however no good simple objective measures exist to explain the functional outcome. Some studies have shown that daily stool frequency and pouch volume are inversely related, yet others recommend an optimal volume around 200 mL [6,7]. Other studies have indicated a relationship between pouch evacuation fraction (EF) and function [8,9,10].

Another reason for high stool frequency could be differences in the small intestinal transit time. Only few studies with oral administration of tracer have investigated transit time in IPAA patients and mainly with a radiopaque marker or barium [11,12,13]. Scintigraphic assessment of the transit time has been investigated in only a few minor studies [14,15]. A reliable method for measurement of small intestinal transit time would be clinically relevant, since subjects with rapid transit could be assumed to benefit from pharmacological intervention.

The aim of this study was to evaluate the feasibility of a clinical simple scintigraphic method for measurement of small intestinal transit time and pouch evacuation in IPAA patients and to investigate any relation with pouch function and QoL.

## 2. Experimental Section

We conducted a cross-sectional study in a Danish University Hospital centre (Odense, Denmark). The study was carried out in accordance with the Declaration of Helsinki and with approval by the National Committee on Health Research Ethics and the Danish Data Protection Agency (Copenhagen, Denmark).

Our goal was to include 20 adult patients with histological diagnosis of ulcerative colitis followed by proctocolectomy and reconstruction of an IPAA. Letter for invitation was sent to 40 randomly selected patients with a functioning IPAA. All patients had a J-pouch design and both patients operated with laparoscopic assisted and open IPAA surgery were included. The staging of IPAA was defined as: (1-stage) proctocolectomy and IPAA in one operation without diversion, (2-stage) proctocolectomy and IPAA with faecal diversion and later stoma closure, (3-stage) subtotal colectomy, IPAA and closure of diverting stoma in three different operations. Patients with co-morbidity contraindicating endoscopy or pregnancy were excluded.

All patients gave written informed consent prior to examinations.

### 2.1. Questionnaires

Pouch function in terms of stool frequency and incontinence was evaluated by a self-reported questionnaire. Minor and major incontinence were defined as seepage and stool incontinence, respectively. QoL was estimated by a validated questionnaire, the Short Inflammatory Bowel Disease Questionnaire (SIBDQ) ranging from 10 to 70 were 70 represents optimal QoL [16,17].

### 2.2. Inflammation

Inflammation was evaluated by endoscopy, pouch biopsy and faecal level of calprotectin. Histology was evaluated by an experienced pathologist and inflammation was defined as polymorphonuclear infiltration of lamina propria, and divided in mild, moderate and severe depending on the severity and presence of crypt abscesses.

### 2.3. Scintigraphy

Two separate scintigraphic examinations were performed in each enrolled patient: a defaecography and an investigation of the intestinal transit time. The overall effective radiation dose was 2.3 mSv for each patient.

**Figure 1:**
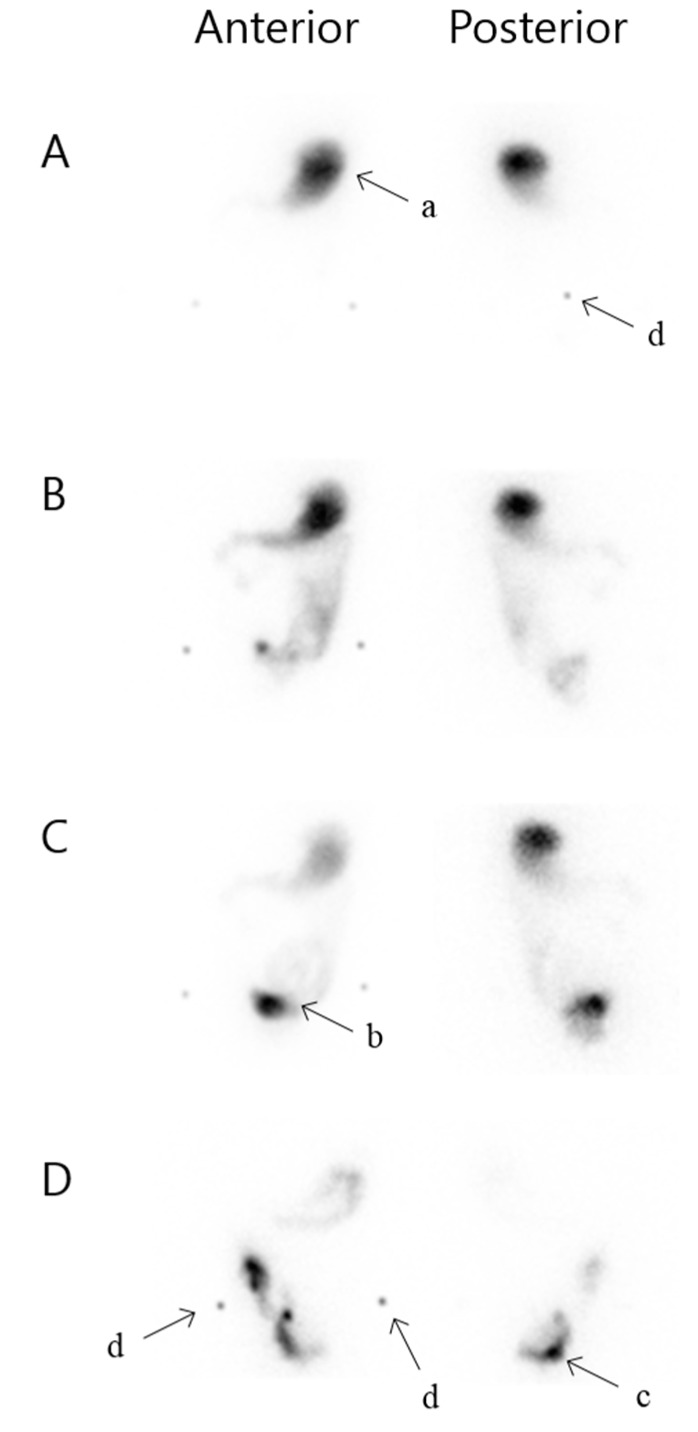
Scintigraphic small intestinal transit time. Anterior and posterior projection. (**A**) 0 min, (**B**) 75 min, (**C**) 105 min, (**D**) 360 min; a: Stomach, b: Loop of small intestine, c: Pouch, d: Anatomic markers, in terms of the anterior superior iliac spine and os coccyx.

Intestinal transit time was determined after oral administration of an omelette labelled with 50 MBq technetium-99m (Tc-99m) together with 200 mL of water. The caloric content of the 100 g omelette was 1400 kJ with 60% carbohydrate, 20% fat, and 20% protein. Abdominal scintigrams of 2 min were obtained with a dual headed gamma camera equipped with LEHR collimators, matrix 128 × 128, in both anterior and posterior projections every 15 min until the tracer was visually cleared from the stomach and the majority of the activity had reached the pouch (Figure 1). This was determined qualitatively with help from anatomic markers and acceptable number of images in every patient. The first acquisition began within 5 min of completing the meal. To obtain alignment of scintigrams and determine anatomic landmarks the anterior superior iliac spine and os coccyx were marked on each image with a tracer pen. The patients were supine during the recordings and were allowed to sit, stand and walk in between. They were fasting 6 h prior to the investigation and ingestion of food and liquid was allowed 3 h after ingestion of the tracer-labelled omelette, and only one patient declined this offer. All medication was taken habitually prior to and during the study. These actions were not standardized, as individual habitual physiological measurements were desired.

Modelling of the intestinal transit time. The method described in the present study was inspired by the convolution method by Brinch *et al.* [18]. Regions of interest (ROIs) over the stomach, small intestine, and pouch were drawn manually on all time frames based on visual assessment of sequential scans. It was defined as visible radioactivity within the small intestine. Geometric mean images were calculated to partially eliminate tissue attenuation. Time activity curves were derived from the geometric mean images and decay corrected. The same physicist performed all analyses.

The time activity curve $ROI_{small\;intestine}$ of the small intestine was modelled individually by convolution of the input curve from the stomach s(t) with a small intestinal impulse response function f(t), see equation (1)

(1) $$ROI_{small\;intestine}\left(t\right)≅\left(f*s\right)\left(t\right)=\int_{0}^{\infty }f\left(\tau \right)s\left(t-\tau \right)d\tau$$

The small intestinal impulse response function f(t) is the probability [0;1] that a given input remains in the small intestine after a time period *t*. The small intestinal impulse response function f(t) was assumed to be a dimensionless smooth step function

(2) $$f\left(t\right)=\frac{1+e^{-\alpha /\beta }}{1+e^{\left(t-\alpha \right)/\beta }}$$

When activity enters the small intestine a spectrum of different transit/residence times occurs. The α parameter (time) describes the point in time where activity begins to leave the small intestine and β (time) is a measure of deviation between transit times, a large β value yields a very narrow spectrum of transit times.

The input function from the gastric emptying s(t) (counts/time) is calculated as the amount of tracer that has left the stomach in the period dt

(3) $$s\left(t\right)=-\frac{dROI_{gastric}\left(t\right)}{dt}$$

The mean transit time ${\bar{t}}_{si}$ of the small intestine is the integral of the time activity curve of the small intestine divided by the initial activity $A_{0}\;$(counts)

(4) $${\bar{t}}_{si}=\;\frac{\int_{0}^{\infty }ROI_{small\;intestine}\left(t\right)dt}{A_{0}}$$

Calculating the mean intestinal transit time from the complete activity curve $ROI_{small\;intestine\;}$ requires knowledge of the entire activity curve, which often requires to image beyond 6 h. Instead we simply calculate the mean transit time ${\bar{t}}_{si}$ from the time integral of the impulse response function f

(5) $${\bar{t}}_{si}=\;\frac{\int_{0}^{\infty }ROI_{small\;intestine}\left(t\right)dt}{A_{0}}\;≅\;\frac{\int_{0}^{\infty }\left(f*s\right)dt\;}{A_{0}}=\;\frac{\int_{0}^{\infty }s\left(t\right)dt}{A_{0}}\int_{0}^{\infty }f\left(t\right)dt=\int_{0}^{\infty }f\left(t\right)dt$$

The parameters α and β describing the impulse response function f was calculated by means of minimizing the sum of least squares between the measured curve $ROI_{small\;intestine}\left(t\right)$ and the calculated convolution curve (f*s)(t) in equation (1). Due to attenuation differences between stomach and abdominal regions [19] a small scaling factor was included in the fitting procedure.

The gastric mean transit time ${\bar{t}}_{gastric}\;$was calculated as the integral of the time activity curve of the stomach

(6) $${\bar{t}}_{gastric}=\frac{\int_{0}^{\infty }ROI\;dt}{A_{0}}$$

### 2.4. Defaecography

On a separate day, a scintigraphic defaecography was performed with per anal instillation of a paste containing water and methyl cellulose in the ratio 350 mL/16 g and labelled with 0.23 MBq Tc-99m-sulphur colloid/mL. Instillation was continued until the patient felt the urge to defaecate and the instilled volume was recorded. Pouch volume was estimated from the instilled paste. A 10 min scintigraphic acquisition with a dual headed gamma camera equipped with LEHR collimators and matrix 128 × 128. Anterior and posterior images was performed before and after defaecation and repeated after a second defaecation one hour later. Pouch EF was calculated from geometric mean values of counts in ROIs drawn on the scintigrams as (def: defaecation):

(7) $$EF\;A\;=\frac{\left(counts\;in\;ROIs\;before\;def\;A-counts\;in\;ROIs\;after\;def\;A\right)}{counts\;in\;ROIs\;before\;def\;A}\;\times \;100\%$$

(8) $$EF\;B\;=\frac{\left(counts\;in\;ROIs\;before\;def\;A-counts\;in\;ROIs\;after\;def\;B\right)}{counts\;in\;ROIs\;before\;def\;A}\;\times \;100\%$$

### 2.5. Statistics

Variables were analysed descriptively according to data type, *i.e.*, continuous variables were expressed by median and range, categorical variables as frequency counts including respective percentages. Univariate correlations were studied with Spearman’s rank correlation coefficient. Transit time and EF were dependent variables, and *p*-values for correlations were designated *P*_transit_ and *P*_EF_, respectively. A multivariate linear regression model was made adjusting for potential confounders including independent variables selected from clinical relevance. Potential confounders included age, sex, body mass index (BMI), stool frequency, pouch volume, histologic inflammation, and EF. Due to the limited sample size, multivariate analyses were of exploratory nature. A two-tailed significance level of 5% was used. Missing data were handled using list-wise deletion. Stata/IC 13 (StataCorp LP, College Station, TX, USA) was used for statistical analysis.

## 3. Results

### 3.1. Patients and Baseline Characteristics

From January 2012 through May 2013 we included 21 patients well represented in relation to sex, time since IPAA surgery, and complications compared with non-respondents. Due to intercurrent pregnancy one patient did not undergo the additional investigation of transit time; hence, only data from 20 patients were included in the final analyses of this parameter.

Patient characteristics, results from the questionnaires and evaluation of pouch inflammation are given in Table 1. Median time from IPAA operation to participation was 932 days (481–3491). In our patient group 14 (67%) patients were treated with diverting stoma. Early postoperative complication with abdominal sepsis was observed in one patient (5%), whereas five (25%) had complications requiring surgery later. The majority (57%) had a three stage surgical procedure, yet a diverting stoma was omitted in four patients due to technical difficulties with a short ileum mesentery. In the questionnaire five patients (25%) stated occasional consideration of pouch removal, but had never requested a pouch removal.

Type of surgery and postoperative complications did not correlate with EF or transit time (data not shown).

**Table 1:** Patient characteristics and baseline results.

Variable	Descriptive Statistics *n* =21
Sex (M/F)	9/12
Age (years)	41.3 (25.8–67.9)
BMI	23.8 (18.7–36.1) ^1^
T_op-study_ (days)	932 (481–3491)
*Smoking (%)*	
Yes	1 (5)
No	11 (52)
Earlier	9 (43)
Extra intestinal symptoms (%)	10 (48)
Diverting stoma after IPAA operation (%)	13 (62)
Laparoscopically assisted/conventional open approach (%)	8/13 (38/62)
*OP stages (%)*	
1	3 (14)
2	6 (29)
3	12 ^2^ (57)
*Postoperative complications (%)*	
Pouchitis	10 (50)
Ileus requiring surgery	3 (15)
Leakage	1 (5)
Fistula requiring surgery	1 (5)
Perforated ulcer	1 (5)
SIBDQ	55 (22–67)
Stool frequency, total	9 (4–25)
*Incontinence, minor and major (%)*	6 (29)
Daytime	3 (14)
Night time	6 (29)
Pouch volume (mL)	222.5 (100–360)
*Histology (%)*	
No inflammation	2 (10)
Light inflammation	12 (57)
Moderate-severe inflammation	7 (33)
Faecal calprotectin (mg·kg^−1^)	200.5 (19–3750)

Values are medians (range) unless otherwise indicated. BMI: Body Mass Index. OP-stages: Number of surgical steps in performing the ileal pouch anal anastomosis. Op-study: Time in days from pouch surgery to inclusion in this study. IPAA: Ileal Pouch-Anal Anastomosis. SIBDQ: Short Inflammatory Bowel Disease Questionnaire. ^1^ Only 20 observations; ^2^ Four without a diverting stoma.

### 3.2. Pouch Function, SIBDQ, and Inflammation

The patients had a median (range) 24-hour stool frequency of nine (4–25) and daytime incontinence was seen in three patients (14%), one major incontinence and two minor (data not shown). The median pouch volume was 222.5 mL (100–360) and found to correlate inversely with stool frequency (*p* = 0.017; coefficient = −6.1) in a univariate analysis (data not shown). Median SIBDQ was 55 (22–67) with no intersexual difference. Ten patients (48%) had previously experienced, but none reported symptoms suggestive of pouchitis during the study. Histology showed moderate to severe inflammation in seven (33%) patients and 16 (75%) had a faecal calprotectin above 50 mg·kg^−1^. Stool frequency, pouch volume, and histology were included in both uni-and multivariate analyses and listed in Table 3. SIBDQ, incontinence, and faecal-calprotectin showed no correlation with the scintigraphic results (data not shown).

### 3.3. Scintigraphic Assessments

To simplify the reading, the mean transit time of the individual is only called “transit time” and the “median transit time” refers to the median of all patients.

The median intestinal transit time ${\bar{t}}_{si}$ was 188 min (105–365) and the median β-parameter was 45.5 min. EF for evacuation 10 min after filling and one hour later was 49% (3–77) and 62% (17–98), respectively. All patients were able to defer the urge to defaecate after instillation. Scintigraphic retrograde evacuation into the afferent ileum was observed in 11 patients (52%).

All scintigraphic results are listed in Table 2 including the median transit time ${\bar{t}}_{gastric}$ for gastric emptying and time for 10% of total activity in the pouch.

**Table 2:** Scintigraphic results including 20 patients.

Median Small Intestinal Transit Time (min)	188.5 (105–365)
Evacuation fraction A (%)	49 (3–77)
Evacuation fraction B (%)	62 (17–98)
Median gastric transit time, ${\bar{t}}_{gastric}$ (min)	78 (40–115)
Interval from mouth to 10% in pouch (min)	172.5 (90–270)
Median spectrum of transit times, β (min)	45.5 (16.7–166.7)

Values are medians (range) unless otherwise indicated. Evacuation fraction A is analysed from scintigrams right after defaecation whereas Evacuation fraction B is derived from scintigrams one hour after the first defaecation. Median small intestinal transit time refers to the median of the mean small intestinal transit time from the patients.

The univariate analyses with transit time and EF as dependent variables showed no significant correlations, however, when adjusting for potential clinical confounders, we found a higher BMI to indicate a significantly longer transit (*P*_transit_ = 0.007; coefficient = 10.7). Moreover, stool frequency affected the transit time inversely (*P*_transit_ = 0.02; coefficient = −10.2). In the multivariate analyses with EF as dependent variable we found no significant results.

The variables included in both the univariate and the multivariate analyses are listed in Table 3.

**Table 3:** Selected results from univariate and multivariate analyses.

Variable	Univariate (*n* = 20)				Multivariate (*n* = 20)		
Association with transit time	*R*^2^	Coefficient	95% CI	*P*_transit_	Coefficient	*R*^2^ = 0.70 95% CI	*P*_transit_
Sex	0.06	35.5	−31 to 102	ns	67.5	−5.8 to 140	0.067
Age	0.16	2.6	−0.4 to 5.6	0.081	2.8	−0.6 to 6.2	0.097
BMI	0.18	6.4	−0.2 to 13	0.057	10.7	3.6 to 17.7	0.007
Stool frequency	0.01	1.4	−5.0 to 7.7	ns	−10.2	−18.3 to −2.0	0.02
Pouch volume	0.004	0.01	−0.6 to 0.5	ns	−0.4	−1.0 to 0.2	ns
Histological inflammation—severe	0.08	8.2	−116 to 132	ns	−26.0	−140 to 88	ns
Evacuation fraction A	0.01	−0.4	−2.0 to 1.2	ns	−0.8	−4.7 to 3.2	ns
Evacuation fraction B	0.02	−0.4	−2.1 to 1.2	ns	−0.9	−4.9 to 3.1	ns
Association with evacuation fraction A	*R*^2^	Coefficient	95% CI	*P*_EF_	Coefficient	*R*^2^ = 0.92 95% CI	*P*_EF_
Sex	0.05	9.5	−10.1 to 29.1	ns	9.4	−21.0 to 39.8	ns
Age	0.06	0.5	−0.4 to 1.4	ns	0.5	−0.9 to 1.9	ns
BMI	<0.01	−0.1	2.4 to 2.2	ns	0.7	−2.3 to 3.6	ns
Stool frequency	0.01	−0.3	−2.2 to 1.5	ns	−2.2	−5.2 to 0.9	ns
Pouch volume	0.04	−0.1	−0.2 to 0.1	ns	−0.1	−0.4 to 0.1	ns
Histological inflammation—severe	0.06	16.4	−20.2 to 52.9	ns	3.2	−44.1 to 50.5	ns

Evacuation fraction A is analysed from scintigrams right after defaecation whereas Evacuation fraction B is derived from scintigrams one hour after the first defaecation. In transit time analyses only 20 patients are included, and 21 patients in analyses of evacuation fraction. *P*_transit_: *p*-value between the variables and transit time. *P*_EF_: *p*-value between the variables and evacuation fraction A. BMI: Body Mass Index. ns: Not significant.

## 4. Discussion

Determination of small intestinal transit time by means of a scintigraphic technique offers several advantages. These include the non-invasive procedure and the possibility to adjust the properties of the radiolabelled markers in accordance with various needs (*i.e.*, liquids, solids). The calculation of the transit time is independent of gastric emptying and pouch filling in the method outlined here. Moreover, the transit time can be calculated without complete emptying of the small intestine. The convolution technique was preferred instead of the more simple technique where small intestinal transit is calculated based on time to filling of the terminal ileum. The convolution method is superior in terms of minimizing the effect of variable emptying of the stomach [19].

Further knowledge about the differentiated transit times (mean and spectrum) of the small intestinal can be gained through modelling. This provides the option of characterising the transit time. When using a method based on time to filling of the pouch the transit time of the front of the radiolabelled meal is estimated. This may not reflect the transit time of the whole bulk of meal. The deconvolution principle offers the opportunity to compute the small intestinal impulse response, which is the expected small intestinal time-activity curve if gastric emptying of the radiolabelled meal were instantaneous, which obviously it is not. The small intestinal impulse response yields the spectrum of all possible transit times from which a mean value can be calculated.

The procedures were well tolerated by the patients, clinically applicable and attempted to be as physiologic authentic as possible.

We evaluated transit time and the degree of pouch evacuation in relation to self-reported stool frequency, incontinence, and QoL as well as pouch volume and histologic inflammation. We found no correlation in univariate analyses and in multivariate analyses only BMI and stool frequency were correlated with the transit time.

Stool frequency and incontinence were used as measures for pouch function as in similar scintigraphic pouch studies. Although Öresland score [3] and Cleveland score [4] are examples of tools to estimate pouch function, there is no general agreement on how to define symptoms of pouch function in the literature. However, it is generally accepted that the number of bowel movements is a measure of pouch function. We found a median stool frequency of nine, which is substantially higher than the mean stool frequency showed by Zeeuw *et al.* [1] in a systematic review from 2012. Our patients were invited to participate voluntarily and perhaps a poor function was overrepresented among the responders, in their hope of an improvement. Moreover, there were slight differences between patients enrolled in the review and our population in regard of indication for pouch surgery, anastomosis technique and surgical approach.

We found major and minor incontinence being more frequent at night compared to daytime with incidences of 29% and 14%, respectively. This correlates well with a new study from Belgium, finding nightly and daily incontinence in 39% and 13% respectively [20].

Pouch volume was found to correspond inversely with stool frequency, which corresponds with other studies in the literature [6,7].

Five patients had a SIBDQ score equal to or below 40, though, the median was 55. This is consistent with the literature for this patient group and shows that our group is not invalidated by the life with an IPAA [21,22]. Yet, it is important to remember that SIBDQ is mainly a tool for extracting changes in QoL in relation to disease activity, whereas a single score only qualifies as an approximation.

Pouchitis is experienced in approximately 50% of all IPAA patients with ulcerative colitis as the primary indication for surgery [18,23]. This was also the case in our study; however none of the patients had clinical symptoms compatible with pouchitis in the study period. We did not observe any endoscopic signs of pouchitis; yet, it is interesting that nearly all had histological inflammation in various degrees and even that 33% showed moderate to severe degree of inflammation. The faecal-calprotectin was elevated in the majority of patients and only four (20%) had a value below the Danish threshold of 50 mg·kg^−1^. Nonetheless this was not related to the histological findings; two out of four patients with normal values showed slight inflammation and the other two had moderate to severe inflammation. These unknown inflammatory findings could be contributing to the functional disorders.

### Scintigraphy

The method outlined in this paper was first described in 1999 by Brinch *et al.* [18] who made studies on individuals with a healthy colon. Using the method for IPAA patients is not described previously to our knowledge. Soper *et al.* [15] conducted a study in 1989 using NaCl containing ^111^In-DTPA instilled directly into duodenum right after ingestion of a meal. They found the time to first arrival in the pouch to be 178 ± 26 min. Their method involves a duodenal catheter and describes the transit time as the time to first arrival of tracer in the pouch. Their result is very similar to ours. Similar to us they did not find any correlation between the transit time and the stool frequency; nevertheless, they found the two subjects with the highest stool frequency to have the fastest transit. In contrary, the patient with the highest stool frequency in our study had the longest transit time, although this was not the tendency throughout our study. Hence, it may be a random finding or indicate that the explanation of the functional disorders is to be found elsewhere. This theory is strengthening by some patients evacuating before any stool has reached the pouch. The similarity in the results indicates that the method of administration; *i.e.*, ingestion or instillation, is of minor concern; thus, an oral approach may be more clinically applicable.

In another study from 1997, Goldberg *et al.* [14] compared patients with good and poor pouch function, respectively, with a threshold of six bowel movements per 24-hour. They investigated the time to pouch filling and excluded incontinent patients. They showed an overall median stool frequency lower than that in our patients (3–11); but when extracting only data in their “poor-function” group, the frequency was similar to ours. They found no intergroup difference in the transit time, which is confirmed by our study. In contrary with Goldberg *et al.* [14], we motivated the eating of a meal three hours after ingestion of the tracer-labelled omelette. This second meal is suggested in the SNMMI and EANM guidelines [19,23] to minimize prolonged stasis of activity in the terminal ileal reservoir. This could potentially affect the transit time, but probably has minor effect due to the three-hour delay. Only one patient declined a second meal after approximately 3 h, hence the potential effect on the transit time was consistent.

Only a solid tracer was used in this study compared to Soper and Goldberg [14,15], who used a dual tracer technique. The gastric emptying is the most problematic issue when measuring small intestinal transit time using an oral agent. Using a dual tracer minimizes this. The deconvolution method also eliminates the dependency of gastric emptying; hence a liquid tracer was omitted in the present study.

Traditionally, radiologic methods have been used for defaecography, but scintigraphic methods are still used and have advantages in terms of the adaption for various settings (*i.e.*, need of solid faecal-like investigations). Mariani *et al.* [24] concluded in 2008 that scintigraphic defaecography is appropriate for investigating pouch dysfunction with no particular discomfort for the patients.

The EF in other studies is highly diverging (28%–77%) [8,25,26] and variations are found in terms of the type of paste instilled, design of the pouch, and diagnosis of the subjects, which makes a comparison difficult. Other studies have shown that J-pouches compared with W-pouches are not emptied as efficiently and have a tendency towards increased retrograde evacuation, but this is not specified in all studies. A stapled J-pouch is the choice of design and anastomosis today and our study population was homogenous in terms of this and the patient diagnosis. Our studyPouch emptying and motility may not be linked. It is possible to evacuate the pouch before any stool has reached the rectum. This may suggest that the stimulating mechanism for evacuation is outside the rectum.

Despite the lack of positive relations the study provides valuable information on expected EF and transit time in IPAA patients. A systemic review on IPAA complications from 2012 comparing studies before and after year 2000 showed a decline in pouch failure after 2000 [1]. Thus it is important to visualize any changes in scintigraphic measured EF and transit times, as there are no newer studies.

An advantage of our study is the simple clinical method that is well tolerated by the patients. The simple oral ingestion makes it possible to apply the method to other populations. Only one investigator did the scintigraphic analyses in order to ensure standardisation, however, this may also increase the risk of systematic bias. The study was not designed for evaluation of possible intra- and inter-observer variation; this could be interesting in future studies in evaluation of the reproducibility of the results.

The relatively small sample size may be part of the explanation for the discrepancy in the results of the uni- and multivariate analysis, although we believe confounding to be the major contributor. Important associations can also be concealed due to the size. Worth of note, however, the number of patients is in line with other studies in this field [14,15].

## 5. Conclusions

In summary, scintigraphic measurement of intestinal transit time and pouch EF is feasible in a simple clinical setting. Our cross sectional study shows no relation between functional outcome and intestinal transit time. Objective factors of the pouch functionality are still warranted, and maybe the pouch volume or maybe histological changes should be investigated more intensely.
